# Chronic Hepatitis C Virus (HCV) Disease Burden and Cost in the United States

**DOI:** 10.1002/hep.26218

**Published:** 2013-05-06

**Authors:** Homie Razavi, Antoine C ElKhoury, Elamin Elbasha, Chris Estes, Ken Pasini, Thierry Poynard, Ritesh Kumar

**Affiliations:** 1 From the Center for Disease AnalysisLouisville, CO; 2 Merck, Sharp, & Dohme CorpWhitehouse Station, NJ; 3 Université Pierre et Marie Curie Liver CenterParis, France

## Abstract

Hepatitis C virus (HCV) infection is a leading cause of cirrhosis, hepatocellular carcinoma, and liver transplantation. A better understanding of HCV disease progression and the associated cost can help the medical community manage HCV and develop treatment strategies in light of the emergence of several potent anti-HCV therapies. A system dynamic model with 36 cohorts was used to provide maximum flexibility and improved forecasting. New infections incidence of 16,020 (95% confidence interval, 13,510-19,510) was estimated in 2010. HCV viremic prevalence peaked in 1994 at 3.3 (2.8-4.0) million, but it is expected to decline by two-thirds by 2030. The prevalence of more advanced liver disease, however, is expected to increase, as well as the total cost associated with chronic HCV infection. Today, the total cost is estimated at $6.5 ($4.3-$8.4) billion and it will peak in 2024 at $9.1 ($6.4-$13.3) billion. The lifetime cost of an individual infected with HCV in 2011 was estimated at $64,490. However, this cost is significantly higher among individuals with a longer life expectancy.

**Conclusion:**

This analysis demonstrates that US HCV prevalence is in decline due to a lower incidence of infections. However, the prevalence of advanced liver disease will continue to increase as well as the corresponding healthcare costs. Lifetime healthcare costs for an HCV-infected person are significantly higher than for noninfected persons. In addition, it is possible to substantially reduce HCV infection through active management.

According to estimates from the National Health and Nutrition Examination Survey (NHANES), 1.6% of the US population was infected with the hepatitis C virus (HCV) in 1999-2002.^1^ In a recent study, over 15,000 deaths were attributed to chronic hepatitis C virus (HCV) infection in 2007,^2^ already exceeding earlier estimates.^3^ HCV infection is associated with chronic, progressive liver disease. Chronic hepatitis C is a leading cause of cirrhosis and hepatocellular carcinoma (HCC),^4^,^5^ which are major indications for liver transplantation.^6^ A better understanding of HCV disease progression and the associated baseline cost, which excludes the cost of antiviral treatment, can help the medical community manage HCV and develop treatment strategies in light of the emergence of several potent anti-HCV therapies.

Historically, researchers have studied HCV disease progression and cost using Markov models.^3^,^7^–^14^ In these models, a homogenous cohort of HCV-infected individuals are introduced, and the model is used to track their progression and cost over time. A recent study^15^ varied the age at infection, gender, and disease duration over time using six cohorts to estimate future disease burden. However, in a previous analysis^16^ it was found that the predictability of the HCV epidemiology model is very sensitive to the number of age and gender cohorts used, due to the large difference in new infections' incidence and mortality across cohorts. Thus, we set out to create a disease progression and cost model that was more refined than those used in previous studies.

The present study represents an improvement over previous work. A total of 36 cohorts composed of 17 5-year age cohorts and one age cohort for 85+ was used for each gender. A system dynamic model was developed to provide maximum flexibility in changing inputs (incidence rate, age at infection, background mortality, transplantation rate, treatment rate, and cost) over time. Finally, more recent healthcare cost data^17^ were used to estimate the HCV cost burden as compared to previous studies that relied on older data.^18^

The goal of this study is to describe the future disease and cost burden of HCV infection in the United States using a systems approach, assuming there is no incremental increase in treatment as the result of the new therapies.

## Materials and Methods

A system dynamic modeling framework was used to construct the model in Microsoft Excel (Redmond, WA) to quantify the HCV-infected population, the disease progression, and the associated cost from 1950-2030. Uncertainty and sensitivity analyses were completed using Crystal Ball, an Excel add-in by Oracle. Beta-PERT distributions were used to model uncertainty associated with all inputs. Sensitivity analysis was used to identify the uncertainties that had the largest impact on the peak cost in 2025. Monte-Carlo simulation was used to determine the 95% confidence interval (CI) for cost and prevalence. When historical data were available, nonlinear polynomial extrapolation of historical data was used for future assumptions in 2012-2030. The Excel optimization add-in, Solver, was used to calibrate the model using reported National Health and Nutrition Examination Survey (NHANES) prevalence data^1^ as described below:


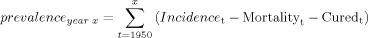


**Fig. 1:**
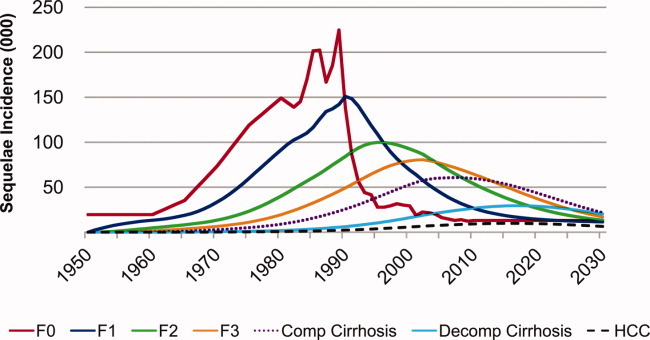
HCV sequelae incidence: US 1950-2030.

Populations in a given health state (incident HCV, cured, F1, F2, etc.) were handled as stocks, while annual transitions from one health state to another were treated as flows with an associated rate/probability (see Supporting Appendix A, Fig. 1). Historical data reporting the number and indications for liver transplantations from 1988 to 2010 were used to estimate the number of transplantations attributable to chronic HCV infection.^6^ Trended transplantation rates from 1988-2011 were used for 1971-1987 and 2011-2030.

The populations were tracked by age cohorts and gender. Five-year age cohorts were used through age 84, and those aged 85 and older were treated as one cohort. Each year, one-fifth of the population in each age group, except for 85 and older, was moved to the next age cohort to simulate aging, after accounting for mortality.

The model started in 1950 to track the prevalent population from the time of infection and forecasted the sequelae populations to 2030. The impact of individuals infected with HCV prior to 1950 was expected to be small and within the margin of error of our analysis. Prevalence of chronic HCV in any given year was calculated by the sum of viremic incidence of new infections (incidence) minus mortality and cured cases, up to that year, as shown below.

Annual background mortality rates by age and gender^19^ were adjusted for incremental increase in mortality due to injection drug use (IDU) and transfusion.^16^ These rates were applied to all populations. For individuals with decompensated cirrhosis (diuretic sensitive and refractory ascites, variceal hemorrhage, and hepatic encephalopathy), HCC, and those who required a liver transplantation, a separate mortality rate was also applied for liver-related deaths,^8^,^10^,^20^ as shown in Supporting Appendix A, Tables 1, 2.

The number of cured patients in 2002-2007 was estimated using published data for the number of treated patients^21^ and an average sustained viral response (SVR) of 34%, as shown in Supporting Appendix B, Table 1. The number of cured patients prior to 2002 was ignored. The number of patients cured in 2008-2030 was extrapolated using 2002-2007 data. The objective of this analysis was to estimate the HCV disease progression and the associated cost in the US when there was no incremental increase in treatment as the result of the new therapies. The launch of direct-acting antivirals in 2011, the increased number of treated patients, and the higher SVR of new therapies were not incorporated in this model. The impact and cost of new therapies were specifically excluded in order to establish a baseline for future comparisons. This, however, will lead to higher projections of advanced liver diseases and poor outcomes as compared to the real world.

With known annual mortality and cured population, annual incidence was calculated using a constant multiplied by the relative incidence. Relative incidence was calculated from the literature data^15^ by dividing each year's incidence by the 1950 incidence to result in a relative incidence of 1 in 1950, as shown in Supporting Appendix C, Table 1. Incidence in the US peaked in 1989 when it was 11.5 times higher than incidence in 1950. Solver was used to find the constant that resulted in a prevalence of 3.2 (95% CI, 2.7-3.9) million in 2000.^1^

The annual incidence was distributed among different age and gender cohorts using distributions reported by the Centers for Disease Control and Prevention (CDC)^22^–^25^ from 1992-2007. Incidence distribution from 2007 was used for 2008-2030 based on the assumption that the future risk factors will remain the same. In 1967-1991, the incidence distribution by age and gender was changed every 5 years and the rates within each 5-year period (e.g., 1967-1971) were extrapolated linearly by age cohort and gender. The distribution was kept constant prior to 1966 based on the assumption that the risk factors remained the same. Solver was used to calculate the annual age and gender distributions, which minimized the difference between the forecasted prevalence age and gender distribution in 2000 and those reported by NHANES.^1^

Since the objective of this study was to determine healthcare costs associated with HCV infection, incremental costs derived from a matched cohort study were used. The cost by sequelae data came from previously published work by McAdam-Marx et al.^17^ The healthcare costs among chronic HCV individuals in F0-F3 stages were adjusted for the proportion not under care (see Supporting Appendix D). The 1950-2010 costs were inflation-adjusted using the Medical Care Services component of the Consumer Price Index.^26^ The 2011 annual medical inflation rate of 3.06% (2.88%-5.33%) was used to estimate future costs in 2012-2030.

The lifetime cost of an HCV-infected individual by age and gender was calculated by introducing 1,000 viremic incident cases in 2011 and using the model to track the progression of these cases and the annual cost over time. The annual healthcare costs for all sequelae and all years were summed and divided by 1,000 to calculate the individual cost. The average cost was calculated by distributing 1,000 new viremic incident cases using 2010 incidence age and gender distribution.^27^

## Results

The annual background and liver-related mortality are shown in Supporting Appendix E. Background mortality is forecasted to peak at 39,935 in 2022 as the HCV population ages, while liver-related deaths peak at 29,695 in 2019 as the number of deaths from decompensated cirrhosis reach their maximum.

Relative incidence and estimated incidence are shown in Supporting Appendix C, Table 1. The constant multiplier for incidence was estimated at 23,790 (20,070-28,990), resulting in a prevalence of 3.2 (2.7-3.9) million in the year 2000.^1^ Incidence values represent acute cases, and 82% (55%-85%)^28^ of these cases progressed to chronic HCV with a METAVIR score of F0, as shown in Supporting Appendix A, Table 1. Incidence for all sequelae is shown in Fig. 1.

**Fig. 2:**
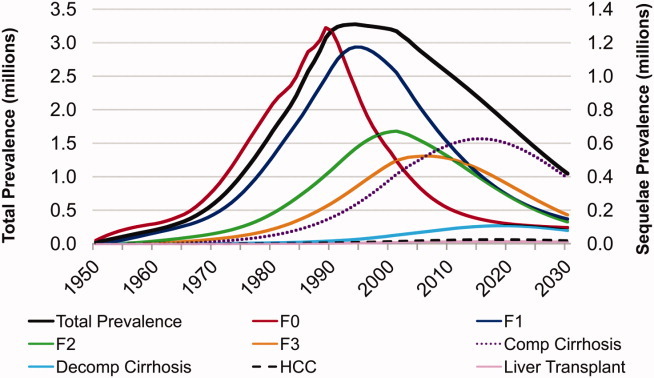
HCV sequelae and total prevalence (millions): US 1950-2030.

Peak viremic prevalence of chronic HCV infection was reached in 1994 with 3.3 (2.8-4.0) million infected individuals (Fig. 2). While the overall prevalence has been declining since, the prevalence of more advanced liver diseases has been increasing. The prevalent population with compensated cirrhosis is projected to peak in 2015 at 626,500 cases, while the population with decompensated cirrhosis will peak in 2019 with 107,400 cases. The number of individuals with HCC, caused by HCV infection, will increase to 23,800 cases in 2018 before starting to decline.

**Fig. 3:**
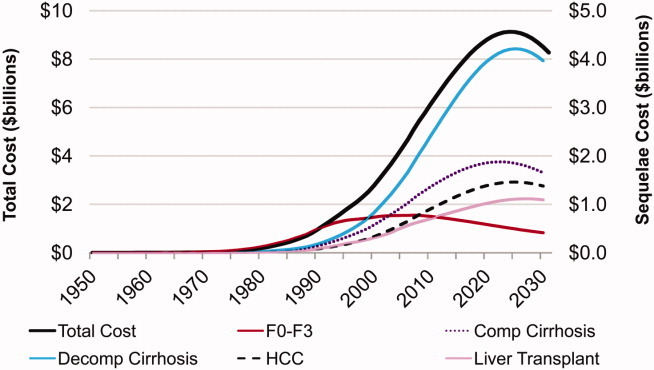
Projected HCV sequelae cost: US 1950-2030.

**Fig. 4:**
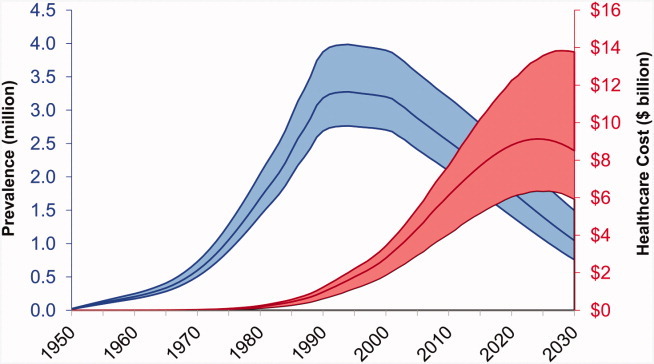
Total prevalence and healthcare costs with 95% CIs.

In 2011, the total healthcare cost associated with HCV infection was $6.5 ($4.3-$8.2) billion. Total cost is expected to peak in 2024 at $9.1 billion ($6.4-$13.3 billion), as shown in Fig. 4. The majority of peak cost will be attributable to more advanced liver diseases—decompensated cirrhosis (46%), compensated cirrhosis (20%), and HCC (16%). The maximum cost associated with mild to moderate fibrosis (F0-F3) occurred in 2007 at nearly $780 million. The cost associated with compensated cirrhosis is expected to peak in 2022 at $1.9 billion, while the peak cost for decompensated cirrhosis and HCC is predicted to occur in 2025, with annual costs in excess of $4.2 billion and $1.4 billion, respectively (Fig. 3).

The lifetime cost of an individual infected in 2011 was estimated at $64,490 ($46,780-$73,190) in 2011 dollars. When medical inflation was applied, the lifetime cost increased to $205,760 ($154,890-$486,890). The lifetime cost estimate varies widely by age and gender due to life expectancy. As shown in Table 1, costs for HCV infections among younger individuals and females will be higher than among the elderly and males.

**Table 1:** Lifetime Cost by Age, HCV Infection, and Gender (in 2011 Dollars)

Age	Male	Female
0-4	$116,600	$147,130
5-9	$105,960	$138,360
10-14	$94,810	$128,440
15-19	$83,430	$117,590
20-24	$76,550	$108,260
25-29	$70,000	$98,040
30-34	$62,950	$87,680
35-39	$57,030	$77,550
40-44	$51,610	$67,880
45-49	$47,180	$59,030
50-54	$35,940	$46,560
55-59	$26,310	$35,510
60-64	$18,540	$26,200
65-69	$12,660	$18,750
70-74	$8,530	$13,170
75-79	$5,630	$9,010
80-84	$3,770	$6,150
85+	$2,680	$4,330

Average - all ages & genders* $64,490 ($46,780 - $73,190)

* Using 2011 incidence age and gender distribution. All values in 2011 dollars with no inflation adjustment.

## Discussion

The predictive value of a model can be confirmed by comparing its forecasts with real-world observations. The model was calibrated using HCV prevalence by age and gender in the year 2000, as reported by NHANES.^1^ The incidence was back-calculated and the model was used to fit reported prevalence in 2000. Total prevalence in other years, prevalence and incidence by sequelae, and mortality were calculated. A 2010 incidence of 16,020 (13,510-19,510) was forecasted versus the reported incidence of 17,000.^29^ The wide CI for incidence was driven by the large uncertainty in reported prevalence.^1^ According to the study by Davis et al.,^15^ HCV incidence peaked in 1989 when it was 11.5 times higher than the incidence in 1950. This corresponded to a peak incidence of 274,000 in a single year. A 2010 prevalence of 2.5 (2.1-3.2) million cases was estimated, matching the most recent NHANES data that showed 2.5 million cases in the 2009-2010.^30^ In comparison, Davis et al.^15^ reported an HCV prevalence of about 3.3 million in the same period.

Our analysis predicted that HCV prevalence in the US peaked in 1994 at 3.3 million viremic cases. The overall prevalence is declining, and the 2030 prevalence is expected to be one-third of the peak prevalence. Incidence has dropped significantly since its peak in 1989 due to the implementation of HCV antibody screening of the blood supply in 1992, with full implementation of universal donation screening for viral RNA through nucleic acid testing (NAT) in 1999,^31^,^32^ and to a decline in IDU.^33^ However, disease burden continues to grow. The dichotomy of HCV is that, while the overall number of infections is projected to decline, the number of individuals experiencing advanced liver diseases, liver related deaths, and healthcare costs are expected to increase. This was a key insight provided by this analysis.

A recent study by the CDC^2^ reported an increased recorded mortality rate in the US HCV-infected population in 1999-2007. Consistent with this study, we forecast that mortality will continue to increase and peak in 2020 (Supporting Appendix E). After 2020, the decline in the number of HCV infections will outweigh the increase in background mortality, and liver-related deaths and the number of deaths will decrease. Mortality is projected to peak at ∼69,440 deaths, with 29,650 deaths attributable to liver disease, including over 9,000 attributed to HCC in 2020.

As shown in Fig. 1, the incidence of more advanced liver diseases will continue to increase, with incidence of decompensated cirrhosis and HCC peaking in 2016-2017. However, not all infected individuals progress to the next stage, and the peak incidence is lower at each consecutive sequelae. The total prevalent population of each sequela is shown in Fig. 2. Over 50% of the HCV prevalent population resides in F0-F3 stage of the disease at any point in time. However, by 2030 compensated cirrhosis cases will account for 37% of all prevalent cases. The HCV compensated cirrhosis population is projected to peak in 2015, while the decompensated cirrhosis population will peak in 2019. A smaller portion of the HCV-infected population will go on to have HCC, but the size of this population does not grow substantially beyond 24,000 due to the very high mortality rate in this population.

A key observation was that peak healthcare costs lag peak prevalence by almost three decades. This is due to the time required for infected cases to progress to more advanced forms of liver disease, which are more expensive to treat.

Sensitivity analysis identified the key drivers of variance in peak healthcare cost. The incidence uncertainty (20,070-28,990), calculated from the uncertainty in NHANES 2000 prevalence, accounted for 52% of the variance in peak cost. Higher incidence led to more prevalent cases and higher cost. Uncertainty in the annual cost of diuretic sensitive ascites ($2,525-$29,860)^17^,^18^ accounted for 15% of the total variance. Finally, uncertainty in persistence (32%-80%)^34^,^35^ accounted for 13% of the variance. Higher persistence resulted in higher SVR and a greater number of cured patients, which in turn resulted in lower healthcare costs. This highlights the importance of SVR on future costs. In this study, the treatment cost was specifically excluded, and yet the SVR of historically treated cases still turned out to be important. The treated population had to be included in the disease progression portion of the model since it affected the size of prevalent populations. In 2002-2011, we estimated that 322,700 individuals were cured. If persistence in the real world were the same as observed in clinical trials (80%),^35^ the average SVR would be 46%, resulting in 430,000 cured cases in 2002-2011. This would result in a decrease of $1 billion dollars in peak healthcare costs.

Patients experiencing decompensated cirrhosis accounted for the majority of future costs. In 2011, it accounted for 40% of total costs, and by 2030 it accounted for 47%. This was followed by compensated cirrhosis (22% of 2011 and 19% of 2030 total cost) and HCC (15% of 2011 and 16% of 2030 total cost). The prevalence of decompensated cirrhosis was 20% of compensated cirrhosis, but the annual cost was 12 times higher.^17^

The average lifetime cost of a patient was estimated at $64,490 as compared to a recent study that reported an average cost of $19,660 per patient in 2002-2010 alone.^17^ The analysis of cost by age at infection demonstrates a link between life expectancy and healthcare cost. Individuals infected in the 1950s were expected to have lower lifetime costs due to lower life expectancy (and lower medical costs), while newly HCV-infected individuals are expected to cost the healthcare systems more due to the longer life expectancy. This highlights the continued importance of prevention as a means of managing future healthcare expenditure.

The effects of new therapies were excluded from our model. However, if the number of treated patients is doubled and kept constant at 126,000 per year in 2012-2030 and the average SVR is increased to 70%, the 2030 prevalent population is projected to be fewer than 100,000 cases. This illustrates that it is possible to substantially reduce HCV infection in the US through active management.

There were a number of limitations in this study that impact the accuracy of our base projections. There is strong evidence that progression transition rates change with age and gender. A single transition rate was used for all ages and genders. This led to a higher incidence/prevalence in early years and among females, as well as higher liver-related mortality among the younger age groups. However, the CIs in our study did capture uncertainty in the above assumptions.

The model does not explicitly account for alcohol consumption and metabolic syndrome. Frequent heavy intake of alcohol significantly increases fibrosis progression,^36^,^37^ and accelerated disease progression has been associated with metabolic syndrome.^38^,^39^ The model implicitly takes these factors into account, as the transition probabilities and sequelae cost incorporate some level of alcohol consumption and metabolic syndrome. If an increasing proportion of the prevalent population experiences heavy alcohol intake or metabolic syndrome, progression to advanced liver disease, and the associated costs, will likely increase.

The model does not take into account the persistent risk of fibrosis progression and liver cancer in virologically cured patients. Observational studies have demonstrated that most patients who achieve SVR experience stabilization or regression of fibrosis. After SVR, episodes of cirrhosis decompensation are extremely rare, and instances of HCC are likely to be small in number and not greatly impact overall disease burden or costs.^40^

A limitation of prevalence measures used in this analysis is that high prevalence populations may be undersampled through the NHANES.^41^ In particular, undersampling of veterans, prisoners, and the homeless would result in underestimation of the current prevalence, future disease, and cost burden. In addition, while IDU has declined from a peak in the 1970s, there is some evidence of a recent increase in IDU among middle-aged adults, potentially leading to a higher incidence of HCV.^33^ In all cases, the sequelae prevalence and the healthcare costs will be higher than the estimated base value.

A further limitation is that the model does not consider recent recommendations^42^ to implement birth cohort screening for HCV. Such screening could reduce the future incidence of advanced liver disease and associated costs, when infected individuals identified through screening receive appropriate treatment and achieve SVR.^43^

Treatment of HCV prior to 2002 was also ignored. The first pegylated interferon was launched in August of 2001, and the number of patients treated with pegylated interferons was small in that year. Prior to that launch, patients were treated with nonpegylated interferon. The number of individuals cured prior to 2001 was small, and their exclusion did not have a material impact on the outcome of the model.

The rate of SVR used in the model was derived from studies of treatment-naïve patients; however, average SVR is lower in treatment-experienced patients. Because the majority of treated patients are naïve, it is unlikely that the use of a single rate for SVR substantially impacted estimates of treated and cured patients beyond our CIs.

A final limitation is that the future cost of liver transplants is based on the assumption that transplantation will remain at the same rate as today. All other sequelae costs were determined as the result of the disease progression. The number of liver transplants, however, is determined by the clinical guidelines and availability of donors. Thus, the future costs associated with liver transplants could be higher if transplantation rates increase.

In conclusion, our analysis demonstrated that overall HCV prevalence in the US is in decline due to lower incidence. However, the prevalence of advanced liver disease will continue to increase, as will the corresponding healthcare costs. Lifetime healthcare costs for an HCV-infected person are significantly higher than for noninfected persons, and the expected cost is higher among populations with a higher life expectancy. Finally, it is possible to substantially reduce HCV infection in the US through active management.

## Abbreviations

CDC: Centers for Disease Control and Prevention
CI: confidence interval
HCC: hepatocellular carcinoma
HCV: hepatitis C virus
IDU: injection drug use
NAT: nucleic acid testing
NHANES: National Health and Nutrition Examination Survey
SVR: sustained viral response.
