# Supplementary material for: Chronic Hepatitis C Virus (HCV) Disease Burden and Cost in the United States
Source: Hepatology. 2013 May 6;57(6):2164–70. doi: 10.1002/hep.26218 (PMC3763475; doi:10.1002/hep.26218)
Supplement: Supplementary file 1 [file hep0057-2164-sd1.rtf]

Appendix A
Figure 1.  Modeled HCV Disease Progression Diagram


Table 1.  Annual Transition Probabilities
	Base	Range	Source	
from Acute (Incidence)	 	 		
% Progress to Chronic HCV	82.00%	(0.85 - 0.55)	(28;44;45)	
from Chronic HCV (F0)	 	 		
…F1	11.70%	(0.104 - 0.13)	(46)	
from F1	 	 		
…F2	8.50%	(0.075 - 0.096)	(46)	
from F2	 	 		
…F3	12.00%	(0.109 - 0.133)	(46)	
from F3	 	 		
…F4 (= Cirrhosis)	11.60%	(0.104 - 0.129)	(46)	
…HCC	0.10%	( 0.0 - 0.002 )	(7;9)	
from Cirrhosis	 	 		
….Diuretic Sensitive Ascites	2.50%	(0.018 - 0.032)	(7;9)	
.…Variceal Hemorrhage	1.10%	(0.006 - 0.016)	(7;9)	
…Hepatic Encephalopathy	0.40%	(0.001 - 0.007)	(7;9)	
…HCC	1.50%	(0.01 - 0.02)	(7;9)	
from Diuretic Sensitive Ascites	 	 		
….Diuretic Refractory Ascites	6.70%	(0.04 - 0.094 )	(7;9)	
….Liver Transplant	See below	 		
….Liver Related Death	11.00%	(0.077 - 0.143)	(7;9)	
from Variceal Hemorrhage	 	 		
….Liver Transplant (1st Year)	See below	 		
….Liver Related Death (1st Year)	40.00%	(0.334 - 0.466)	(7;9)	
….Liver Related Death (Sub Yrs)	13.00%	(0.085 - 0.175)	(7;9)	
from Hepatic Encephalopathy	 	 		
….Liver Transplant (1st Year)	See below	 		
….Liver Related Death (1st Year)	68.00%	(0.659 - 0.701)	(7;9)	
….Liver Related Death (Sub Yrs)	40.00%	(0.378 - 0.422)	(7;9)	
from Diuretic Refractory Ascites	 	 		
….Liver Transplant	See below	 		
….Liver Related Death	33.00%	(0.28 - 0.38)	(7;9)	
from HCC	 	 		
….Liver Related Death (1st Year)	70.70%	(0.43 - 0.77)	(7;9;47)	
….Liver Related Death (Sub Yrs)	16.20%	(0.11 - 0.23)	(7;9;47)	
				
Liver Transplant (1950-1970)	0%	 Transplant rates were negligible	
Liver Transplant (1971-1987)	5.3%	(0.031 - 0.0542)	(7), Trended Data	
Liver Transplant (1988-2010)	Actual Data	33% attributed to HCV	(5) 	
Liver Transplant (2011-2030)	1.7%	(0.0169 - 0.045)	Trended Data	


Table 2.  Liver Transplantation: Liver Related Mortality Rates by Year
	First Year	Std Error	Subsequent Years	Std Error	
1971-1986	33.10%	2.80%	3.87%	7.64%	
1987	33.10%	2.80%	3.87%	7.64%	
1988	24.20%	1.10%	3.64%	2.80%	
1989	24.20%	1.00%	3.94%	2.63%	
1990	21.30%	0.90%	3.84%	2.29%	
1991	20.80%	0.80%	4.11%	2.10%	
1992	19.50%	0.80%	4.28%	2.09%	
1993	17.80%	0.70%	4.16%	1.82%	
1994	15.60%	0.70%	3.85%	1.73%	
1995	15.80%	0.60%	3.93%	1.54%	
1996	15.90%	0.60%	4.06%	1.56%	
1997	14.10%	0.60%	4.02%	1.52%	
1998	14.10%	0.60%	4.02%	1.52%	
1999	14.70%	0.60%	4.22%	1.20%	
2000	13.30%	0.50%	3.91%	1.11%	
2001	14.10%	0.50%	4.05%	1.12%	
2002	13.00%	0.50%	3.97%	1.11%	
2003	13.60%	0.50%	4.03%	1.12%	
2004	12.90%	0.50%	5.19%	0.96%	
2005	13.40%	0.50%	4.85%	0.96%	
2006	12.50%	0.50%	4.85%	0.96%	
2007	10.70%	0.40%	4.85%	0.96%	
2008-2030	10.70%	0.40%	4.85%	0.96%	
Data from Organ Procurement and Transplantation Network was used for 1987-2007. It was assumed that mortality rates in 2008-2030 were the same as 2007. In addition, the mortality rates for 1987 were applied to 1971-1986. (Source:  2009 OPTN/SRTR Annual Report 1999-2008: Table 9.15a. Unadjusted Patient Survival by Year of Transplant at 3 Months, 1 Year, 3 Years, 5 Years and 10 Years, Deceased Donor Liver Transplants Organ Procurement and Transplantation Network (OPTN). Rockville, MD: U.S. Department of Health and Human Services, Health Resources and Services Administration, Healthcare Systems Bureau, Division of Transplantation; 2009.)


Appendix B
Table 1.  Estimated Number of Treated and Cured HCV Patients in the US*
Year	Treated HCV Patients	Cured HCV Patients	
2002	126,040 	43,293	
2003	107,131 	36,798	
2004	144,276 	49,557	
2005	114,197 	39,225	
2006	88,083 	30,255	
2007	82,270 	28,259	
2008	79,301 	27,239	
2009	71,907 	24,699	
2010	63,115 	21,679	
2011-2030	63,115 	21,679 	
*The annual number of treated patients came from previous studies (21). Treated patients in 2008-2010 were extrapolated from the reported numbers in 2002-2007. The number of treated patients in 2011-2030 was kept constant at 63,115, same as 2010. Previous studies suggest that the genotype distribution of the treated population is the same as the prevalent population (34). Thus, it was assumed that 22% of the treated population were genotypes 2/3 (Alter et al., 1999) with a sustained viral response (SVR) of 66% (Manns et al., 2011) and the rest, who are composed mostly of genotype 1, had an SVR of 40% (35), resulting in a weighted average SVR of 46%. In clinical trials, 80% of patients completed treatment (35). Among veterans, only 32% of patients completed treatment (34). However, veterans represent a mostly male population with a higher percentage of drug and alcohol abuse and depression as compared to the US population. A persistence of 60% was assumed for the HCV treated population in the real world, resulting in an average SVR of 34%.
Sources: Alter MJ, Kruszon-Moran D, Nainan OV, McQuillan GM, Gao F, Moyer LA, et al. The prevalence of hepatitis C virus infection in the United States, 1988 through 1994. N.Engl.J.Med. 1999;341:556-562.
Manns M, Zeuzem S, Sood A, Lurie Y, Cornberg M, Klinker H, et al. Reduced dose and duration of peginterferon alfa-2b and weight-based ribavirin in patients with genotype 2 and 3 chronic hepatitis C. J.Hepatol. 2011;55:554-563.


Appendix C
Figure 1.  Relative incidence 1950-2030*

*  Calculated from incidence estimates by Davis, et al. (14). Each year's incidence was divided by incidence in 1950 to calculate the relative incidence over time.


Table 1.  Annual Relative Incidence and Incidence
Year	Relative Incidence	Annual Incidence		Year	Relative Incidence	Annual Incidence	
1950	1.0	23,790		1991	4.4	105,520	
1951	1.0	23,790		1992	2.9	68,780	
1952	1.0	23,790		1993	2.3	53,700	
1953	1.0	23,790		1994	2.1	50,880	
1954	1.0	23,790		1995	1.4	33,920	
1955	1.0	23,790		1996	1.4	33,920	
1956	1.0	23,790		1997	1.5	35,800	
1957	1.0	23,790		1998	1.6	38,630	
1958	1.0	23,790		1999	1.5	36,740	
1959	1.0	23,790		2000	1.5	35,800	
1960	1.0	23,790		2001	1.0	22,610	
1961	1.2	27,650		2002	1.1	27,320	
1962	1.3	31,520		2003	1.1	26,380	
1963	1.5	35,380		2004	1.0	24,500	
1964	1.6	39,250		2005	0.8	19,790	
1965	1.8	43,110		2006	0.8	17,900	
1966	2.2	52,290		2007	0.7	16,020	
1967	2.6	61,470		2008	0.7	16,960	
1968	3.0	70,650		2009	0.6	15,070	
1969	3.4	79,830		2010	0.7	16,020	
1970	3.7	89,010		2011	0.7	16,020	
1971	4.2	100,190		2012	0.7	16,020	
1972	4.7	111,380		2013	0.7	16,020	
1973	5.2	122,570		2014	0.7	16,020	
1974	5.6	133,760		2015	0.7	16,020	
1975	6.1	144,940		2016	0.7	16,020	
1976	6.4	152,310		2017	0.7	16,020	
1977	6.7	159,690		2018	0.7	16,020	
1978	7.0	167,060		2019	0.7	16,020	
1979	7.3	174,430		2020	0.7	16,020	
1980	7.6	181,800		2021	0.7	16,020	
1981	7.4	175,690		2022	0.7	16,020	
1982	7.1	169,590		2023	0.7	16,020	
1983	7.4	177,120		2024	0.7	16,020	
1984	8.7	206,330		2025	0.7	16,020	
1985	10.3	245,900		2026	0.7	16,020	
1986	10.4	246,840		2027	0.7	16,020	
1987	8.6	203,500		2028	0.7	16,020	
1988	9.5	226,110		2029	0.7	16,020	
1989	11.5	274,160		2030	0.7	16,020	
1990	7.1	168,640					


Appendix D
Table 1.  Incremental Cost of HCV Patients by Sequelae (16)*
	PPPY‡ Cost (2011 $) - Base	Range	
HCV (F0-F3) §	$426	($240 - $610)	
Compensated Cirrhosis	$2,375	($1,600 - $3,140)	
Diuretic Sensitive Ascites	$28,130	($2,525† - $29,860)	
Refractory Ascites	$28,130	($26,400 - $29,860)	
Variceal Hemorrhage - 1st Yr	$28,130	($26,400 - $29,860)	
Variceal Hemorrhage - Subseq Yrs	$28,130	($5,165† - $29,860)	
Hepatic Encephalopathy - 1st Year	$28,130	($26,400 - $29,860)	
Hepatic Encephalopathy - Subseq Yrs	$28,130	($3,930† - $29,860)	
Decompensated Cirrhosis	$28,130	($26,400 - $29,860)	
Hepatocellular Carcinoma	$44,870	($40,270 - $49,465)	
Liver Transplant	$178,130	($164,265 - $191,990)	
Liver Transplant - Subseq Yrs	$38,795	($31,700 - $45,890)	
*These costs reflect the incremental cost of HCV patients. The medical cost of patient cohort without HCV has been subtracted from the above numbers. For example, total healthcare cost for the first year of liver transplant was estimated at $203,740 in 2011 dollars. However, the cost among HCV infected individuals was $178,127 higher than for the matched comparison persons in 2011 dollars. In addition, the cost of antiviral therapy, as reported by the McAdam-Marx et al. study (16), was subtracted to reflect the incremental medical costs net of HCV treatment.
†Only cost for all decompensated cirrhosis was reported in the McAdam-Marx et al. study (16). A lower range was used for select indications when significantly lower estimates were reported in the literature (17).
‡PPPY, per patient per year.
§The cost of individuals with chronic HCV infections was adjusted for percentage of patients not under medical care. According to McAdam-Marx et al. study (16), the cost of HCV patients without liver disease is $3,654 per patient in 2011 dollars (net cost of antiviral therapies). However, this cost applies only to patients who were under medical care. According to the same study, 30.4% of these HCV patients had antiviral utilization. According to our model, 3.5% of individuals in F0-F3 stage were treated in 2002-2010. Thus, we estimated that 11.6% (3.5%/30.4%) of all HCV infected (F0-F3) individuals were under medical care in this period. The average medical cost for all HCV infected without liver disease was estimated at $426 ($3,654 times 11.6%) per patient per year (PPPY). 
 


Appendix E
Table 1.  Annual Mortality
		Liver Related Deaths by Sequela			
Year	Background Mortality	Diuretic Sensitive Ascites	Variceal Hemorrhage	Hepatic Encephalopathy	Diuretic Refractory Ascites	HCC	Liver Transplant	Total Liver-related Deaths	Total Deaths	
1950	0	0	0	0	0	0	0	0	0	
1951	210	0	0	0	0	0	0	0	210	
1952	418	0	0	0	0	0	0	0	418	
1953	619	0	0	0	0	0	0	0	619	
1954	813	0	0	0	0	0	0	0	813	
1955	966	0	0	0	0	0	0	0	966	
1956	1,167	0	0	0	0	0	0	0	1,167	
1957	1,371	0	0	0	0	0	0	1	1,372	
1958	1,617	0	0	0	0	1	0	1	1,618	
1959	1,803	0	1	0	0	2	0	3	1,807	
1960	2,005	1	1	1	0	4	0	7	2,012	
1961	2,263	2	2	1	0	7	0	13	2,276	
1962	2,475	4	4	2	1	11	0	21	2,496	
1963	2,827	6	6	4	1	17	0	33	2,860	
1964	3,234	9	9	5	2	24	0	50	3,283	
1965	3,587	14	13	8	3	34	0	72	3,658	
1966	4,034	20	19	11	5	46	0	100	4,134	
1967	4,589	28	25	14	7	60	0	134	4,723	
1968	5,081	37	33	18	10	77	0	176	5,257	
1969	5,824	49	43	23	14	97	0	226	6,049	
1970	6,339	63	54	29	19	119	0	284	6,623	
1971	6,852	79	67	36	25	145	0	351	7,204	
1972	7,358	94	81	43	31	174	17	439	7,798	
1973	8,006	111	97	51	37	206	38	540	8,546	
1974	8,640	131	113	58	45	242	56	644	9,284	
1975	9,034	153	131	67	53	281	73	759	9,793	
1976	9,590	179	152	77	63	325	90	885	10,475	
1977	10,184	208	174	88	74	374	108	1,025	11,209	
1978	10,966	239	200	101	86	427	127	1,180	12,146	
1979	11,849	275	228	115	99	487	149	1,353	13,202	
1980	12,673	314	260	130	115	553	172	1,544	14,217	
1981	13,875	358	295	148	132	627	198	1,756	15,632	
1982	14,502	406	334	167	151	709	226	1,993	16,495	
1983	14,901	460	377	188	172	800	258	2,255	17,156	
1984	15,719	521	426	212	195	902	293	2,548	18,267	
1985	16,874	588	480	239	221	1,015	332	2,874	19,748	
1986	18,356	662	540	268	250	1,140	376	3,236	21,592	
1987	20,245	745	606	301	282	1,279	424	3,638	23,883	
1988	21,411	837	680	338	318	1,433	358	3,963	25,374	
1989	23,180	939	762	378	358	1,602	434	4,473	27,653	
1990	24,701	1,054	852	422	403	1,788	454	4,972	29,672	
1991	25,749	1,174	951	470	450	1,990	535	5,571	31,319	
1992	26,599	1,310	1,059	523	504	2,212	599	6,207	32,806	
1993	27,644	1,465	1,175	579	566	2,452	635	6,872	34,515	
1994	29,381	1,630	1,304	642	633	2,713	648	7,570	36,951	
1995	30,327	1,814	1,445	710	709	2,993	742	8,413	38,740	
1996	30,995	2,014	1,596	783	793	3,292	840	9,319	40,314	
1997	29,971	2,236	1,761	862	886	3,612	839	10,197	40,168	
1998	31,207	2,479	1,938	946	992	3,953	919	11,227	42,435	
1999	31,683	2,737	2,130	1,038	1,106	4,313	1,036	12,361	44,044	
2000	32,148	3,015	2,335	1,134	1,230	4,690	1,036	13,440	45,588	
2001	32,590	3,310	2,550	1,234	1,365	5,082	1,160	14,700	47,290	
2002	32,915	3,623	2,776	1,337	1,511	5,486	1,165	15,898	48,813	
2003	32,967	3,903	3,010	1,443	1,647	5,900	1,285	17,189	50,156	
2004	33,155	4,190	3,207	1,525	1,790	6,255	1,308	18,275	51,430	
2005	33,197	4,453	3,410	1,610	1,926	6,608	1,524	19,531	52,728	
2006	33,348	4,728	3,590	1,682	2,069	6,925	1,497	20,491	53,840	
2007	33,671	5,013	3,774	1,757	2,220	7,240	1,412	21,415	55,086	
2008	34,049	5,304	3,963	1,836	2,376	7,550	1,501	22,530	56,579	
2009	34,489	5,592	4,149	1,912	2,537	7,842	1,565	23,596	58,086	
2010	34,973	5,872	4,330	1,985	2,698	8,111	1,615	24,610	59,584	
2011	35,529	6,145	4,505	2,053	2,860	8,357	1,654	25,574	61,103	
2012	36,104	6,394	4,671	2,115	3,015	8,581	1,691	26,467	62,572	
2013	36,694	6,622	4,822	2,166	3,162	8,773	1,727	27,272	63,966	
2014	37,275	6,824	4,952	2,207	3,299	8,931	1,759	27,972	65,248	
2015	37,835	6,999	5,062	2,239	3,424	9,052	1,786	28,562	66,397	
2016	38,347	7,143	5,151	2,260	3,536	9,137	1,808	29,034	67,382	
2017	38,821	7,256	5,218	2,271	3,632	9,182	1,825	29,383	68,204	
2018	39,227	7,334	5,261	2,271	3,712	9,189	1,837	29,604	68,831	
2019	39,552	7,378	5,279	2,261	3,773	9,157	1,846	29,694	69,246	
2020	39,787	7,386	5,274	2,241	3,816	9,087	1,849	29,652	69,439	
2021	39,917	7,359	5,244	2,210	3,839	8,979	1,847	29,479	69,396	
2022	39,933	7,296	5,190	2,171	3,843	8,835	1,841	29,176	69,109	
2023	39,830	7,200	5,113	2,122	3,826	8,656	1,828	28,747	68,577	
2024	39,599	7,071	5,014	2,066	3,790	8,446	1,811	28,198	67,797	
2025	39,241	6,911	4,894	2,002	3,735	8,206	1,787	27,535	66,776	
2026	38,752	6,723	4,754	1,931	3,662	7,939	1,758	26,766	65,518	
2027	38,133	6,507	4,597	1,854	3,571	7,648	1,723	25,900	64,033	
2028	37,391	6,268	4,423	1,772	3,465	7,336	1,683	24,947	62,338	
2029	36,529	6,009	4,236	1,686	3,344	7,007	1,637	23,918	60,447	
2030	35,553	5,731	4,037	1,597	3,210	6,664	1,586	22,824	58,378	
