# Supplementary figures and images for: Chronic Hepatitis C Virus (HCV) Disease Burden and Cost in the United States
Source: Hepatology. 2013 May 6;57(6):2164–70. doi: 10.1002/hep.26218 (PMC3763475; doi:10.1002/hep.26218)

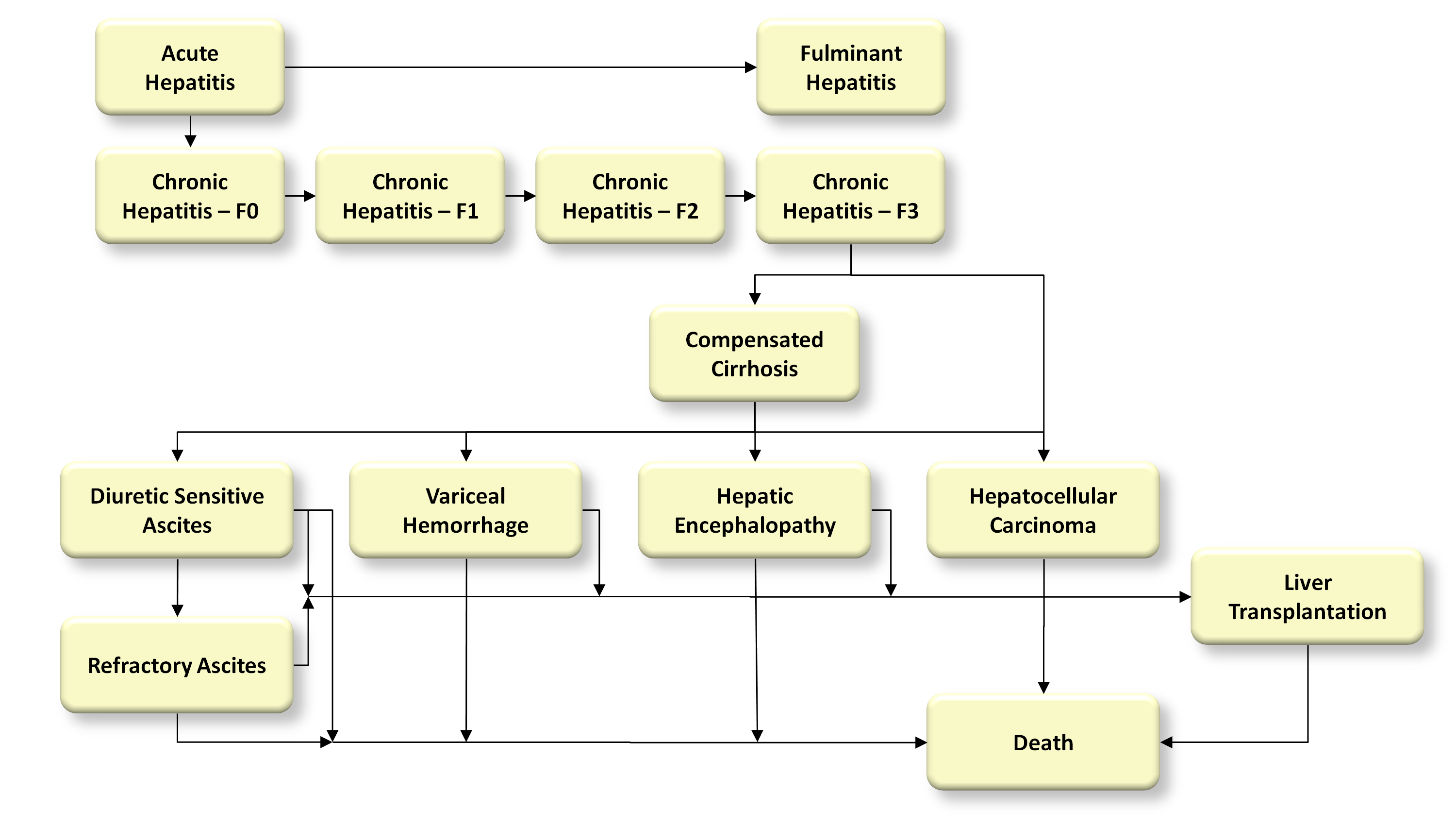

Supplement: Supplementary file 2 [file hep0057-2164-sd2.tif]

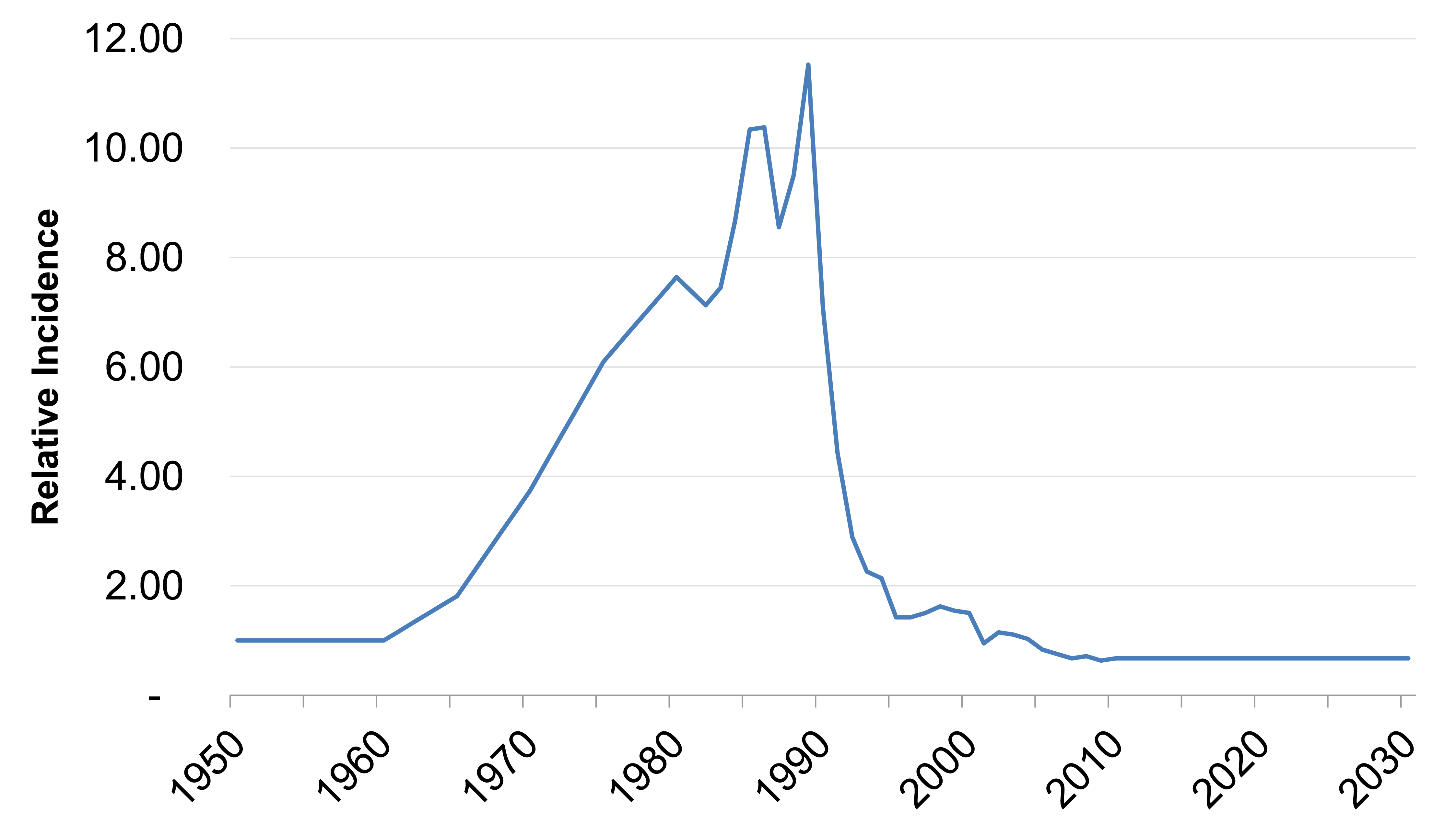

Supplement: Supplementary file 3 [file hep0057-2164-sd3.tif]
